# Nurse Staffing, Work Hours, Mandatory Overtime, and Turnover in Acute Care Hospitals Affect Nurse Job Satisfaction, Intent to Leave, and Burnout: A Cross-Sectional Study

**DOI:** 10.3389/ijph.2024.1607068

**Published:** 2024-04-30

**Authors:** Sung-Heui Bae

**Affiliations:** College of Nursing, Graduate Program in System Health Science and Engineering, Ewha Womans University, Seoul, Republic of Korea

**Keywords:** nurse staffing, work hours, mandatory overtime, job satisfaction, intent to leave, burnout, hospitals, cross-sectional study

## Abstract

**Objectives:** This study examined the impact of nurse staffing, working hours, mandatory overtime, and turnover on nurse outcomes in acute care hospitals. Previous studies have focused on the single characteristics of sub-optimal nurse staffing but have not considered them comprehensively.

**Methods:** Data were collected in July–September 2022 using convenience sampling and an online survey (*N* = 397). For the analysis, 264 nurses working as staff nurses at 28 hospitals met the inclusion criteria. Univariate analysis and multivariable generalized estimating equation (GEE) were performed.

**Results:** Both nurse staffing (β = −0.036, standard error [SE] = 0.011) and turnover (β = −0.006, SE = 0.003) were significant factors affecting job satisfaction. In the multivariable GEE, only mandatory overtime (β = 0.395, SE = 0.116) was significantly related to intent to leave. Nurse staffing, work hours, mandatory overtime, and turnover were not significantly related to burnout. Subjective health status and workload were significantly associated with burnout.

**Conclusion:** Nurse staffing policies and improvement programs in hospitals should be implemented to improve nurses’ job satisfaction. Labor policy should ban mandatory overtime.

## Introduction

Nursing shortage is a global issue that many countries experience in their healthcare systems [1]. The World Health Organization [2] estimated that the world encountered a shortage of 5.9 million nurses in 2018. The International Council of Nurses projects that over 13 million nurses will be required to resolve nursing shortages by 2030 [3]. The COVID-19 pandemic has enhanced the demand for nurses and exacerbated the shortage [4]. Nurse turnover rates are as high as 27.65% and 23% in the United States [5] and Israel [6], respectively. In South Korea, the number of licensed registered nurses per 1,000 population was 8.9 in 2021, and approximately half of them (4.6 nurses per 1,000 people) worked as clinical nurses [7]. These numbers are below average for other high-income countries (e.g., Organization for Economic Co-Operation and Development countries) [7]. The nurse turnover rate in South Korea is 15.2%, and the turnover rate of newly licensed nurses is 44.5% [8].

Sub-optimal nurse staffing characteristics can be observed during nursing shortages, such as inadequate staffing levels, longer working hours with overtime, and high turnover [9]. Several systematic reviews and meta-analyses have reported on the relationship between nurse staffing and patient outcomes. For example, sub-optimal nurse staffing has an adverse impact on the quality of care. Specifically, nurse staffing is significantly associated with patient mortality [10] and hospital-acquired conditions, including pressure ulcers, falls, central line-associated bloodstream infections, and catheter-associated urinary tract infections [11]. Long working hours are adversely associated with quality of care, patient safety, errors, patient satisfaction, and patient mortality [12]. A recent review found that turnover decreases patient satisfaction, while increasing pressure ulcers, and medication errors [13].

Regarding nurse outcomes, inadequate nurse staffing increases nurses’ burnout, job dissatisfaction, and intent to leave [14]. Among nurses working in critical care units, inadequate staffing also increases burnout, fatigue, stress, job dissatisfaction, and plans to leave [15]. Long work hours were significantly associated with various nurse outcomes, such as occupational injuries, absenteeism burnout, job dissatisfaction, intent to leave, fatigue, and overweight/obesity, while mandatory overtime increased injury, illness, and absenteeism [16]. Relatively few studies have examined turnover and nurse outcomes [13]; however, turnover decreases nurses’ mental health and job satisfaction [17].

The aforementioned studies have often focused on a single characteristic of sub-optimal nurse staffing, instead of the various nurse staffing characteristics, such as lower staffing levels, long work hours, and high turnover, which often occur concurrently during nursing shortages. A previous study [9] investigated comprehensive nurse staffing characteristics to examine the relationship between these nurse staffing characteristics and patient outcomes, not nurse outcomes. Another study [18] examined work-schedule and its associations with burnout and intention to leave among nurses working in psychiatric hospitals. However, these sub-optimal nurse staffing characteristics including staffing levels, work hours, and turnover have not been comprehensively considered when investigating their relationship with nurse outcomes in acute care hospitals. During nursing shortages, such sub-optimal nurse staffing characteristics can lead to poor nurse outcomes, including lower job satisfaction and high burnout, which, in turn, can influence additional nurse turnover and aggravate the nursing shortage. Thus, understanding which staffing characteristics are stronger contributing factors to specific nurse outcomes would provide more informative evidence for developing strategies and policies to improve these nurse outcomes and eventually reduce turnover and retain nurses given nursing shortages.

Based on Donabedian’s structure, process, and outcomes model [19], these sub-optimal nurse staffing characteristics can be structural aspects that affect nurse outcomes through processes. This study focused on the structure and outcomes. According to previous studies, specific variables of sub-optimal nurse staffing include nurse staffing levels, work hours, mandatory overtime, and turnover [9, 18]. Similarly, previous studies indicate that nurse outcomes related to these sub-optimal nurse staffing characteristics include job satisfaction, intent to leave, and burnout [14, 16, 17]. Therefore, using Donabedian’s model and findings from previous studies, this study examined the impact of sub-optimal nurse staffing characteristics on nurse outcomes including job satisfaction, intent to leave, and burnout, in acute care hospitals.

## Methods

### Study Design and Sample

This cross-sectional study examined the impact of nurse staffing, work hours, mandatory overtime, and nurse turnover on nurse outcomes using data collected from nurses working in the medical and surgical nursing units of acute care hospitals. According to G*Power 3.1.9.4 [20], a minimum of 123 participants was required for a multivariable generalized estimating equation (GEE) with 11 predictors, 0.15 effect size, a significance level of 0.05, and a power of 0.80. Convenience sampling was used for data collection and was conducted from July to September 2022. Nurses who worked in the current unit for 6 months or longer and provided direct patient care were included.

### Data Collection

For data collection, small- and medium-sized general hospitals in South Korea were contacted to explain the study and invite them to participate. The author delivered invitation information with the online survey link, which was sent to staff nurses and nurse managers within each hospital. Using the online survey, data confidentiality and anonymity were maintained for all participants. At the end of the survey, all participants left their mobile number to receive compensation in the form of a small gift. Of the 270 general hospitals with 201–1,000 beds, 35 agreed to participate. A total of 397 nurses and nurse managers from these hospitals responded to an online survey. Inclusion criteria included working in either a medical, surgical, or medical-surgical combined unit and working in the current unit for 6 months or more. A total of 45 and 29 participants, respectively, were excluded for not meeting these criteria. In addition, 26 participants who did not answer more than 70% of the questions were excluded. Finally, 33 nurse managers who did not provide direct patient care were excluded. The final analytical sample comprised 264 registered nurses from 28 hospitals who worked as staff nurses, which was sufficient for the multivariable GEE.

### Measures

#### Dependent Variables

The nurse outcomes included job satisfaction, intent to leave, and burnout. The Copenhagen Psyco-Social Questionnaire Scale [21, 22] was used to measure job satisfaction. This scale comprises four items rated on a four-point Likert scale. The exam item was, “To what extent are you satisfied with your career prospects?” The total mean score ranged from 1 to 4 points, and higher scores indicated greater job satisfaction. The Intraclass correlation coefficient (ICC (1)) was 0.071. The Cronbach’s alpha for this study was 0.86, indicating good internal consistency.

Turnover intention [23] was used to measure nurses’ intent to leave. Park et al. [24] modified this original instrument for nurses and used it in this study. The instrument comprised four items with a five-point Likert-type scale. The exam item was “I sometimes think of leaving my current workplace.” Higher scores indicated greater turnover intention, and the total mean score ranged from 1 to 5 points. ICC (1) of turnover intention was 0.035. Good internal consistency was found in this study (Cronbach’s alpha = 0.86).

Nurse burnout was measured using the Professional Quality of Likes Scale (ProQOL version 5) [25, 26]. In the ProQOL, burnout under the compassion satisfaction/fatigue subscale was used, which comprises 10 items rated on a five-point Likert-type scale. The total burnout scores ranged from 10 to 50. Higher scores indicated higher levels of burnout. A score of 22 or lower is considered “low,” and a score between 23 and 41 is considered “moderate.” A score of 42 or higher is considered “high [25]”. The ICC (1) of burnout was 0.067. Cronbach’s alpha was 0.76 for this study, indicating acceptable internal consistency.

#### Main Independent Variables

The nurse staffing level was measured as the number of patients per nurse during a shift. Nurses working a three-rotating shift (day, evening, and night) provided the number of patients during each shift. The average number of patients per nurse was used to measure the nurse staffing levels. Other nurses reported the number of patients during the shift. Nurses recalled their staffing levels during the previous month. When nurses did not provide the number of patients during a shift, they provided the number of beds and the total number of nurses in their unit. Using these data, the nurse staffing level during a shift (nurse to patient ratio) was calculated by the number of beds per the total number of nurses multiplied by 4.8, which was based on an assumption of 226 working days per year for nurses working 3 shifts (3 × 365/226 = 4.845) [27]. For example, when the number of beds in a unit was 60 and the total number of nurses in that unit was 20, then the nurse staffing level during a shift was 14.4 (60/20 × 4.8 = 14.4) for the three-rotating shifts. This method has been used previously [27]. All staffing data were manually reviewed. Outliers were also checked such as unusually high or low numbers. Among 264 nurses, 7 cases were missing, and 4 cases were the outliers and imputed as missing. The nurse staffing level (the number of patients per nurse during a shift) were used for 164 nurses, and the number of beds per the total number of nurses multiplied by 4.8 were used for 89 nurses. Sensitivity analysis was conducted to evaluate the validity of this method. The GEE models were run both with (253) and without (164) the cases. The estimates of nurse staffing remained stable, showing no difference in the significant levels or direction. The strength of estimate was similar in the job satisfaction and burnout models and a little different in the intent to leave model. The staffing calculation was found to be valid.

Nurses’ work hours were defined as the mean shift length. Actual work hours were measured for day, evening, and night shifts among nurses working in a three-rotating shift. Other nurses reported the number of working hours per shift. The average actual work hours in the previous months were used. Unusually long work hours, such as outliers, were considered errors and imputed as missing data. Nurses also answered whether they had worked mandatory overtime in the previous months (yes/no), based on their perception of mandatory overtime.

Nurse turnover rates were measured using the prior six-month unit turnover rates. The nurse managers provided data on nurse turnover. Data on the number of nurses who worked at the unit and left it between 1 January 2022, and 30 June 2022 (approximately 6 months prior to data collection) were collected. The denominator is the average number of nurses working between 1 January and 30 June, 2022, and the numerator is the number of nurses who resigned during the same period. The turnover rate was calculated for nurses working with nurse managers in the same unit [28]. This six-month turnover rate was used in the analysis.

#### Covariates

Nurses’ characteristics included sex, age, the highest level of nursing education, marital status, and subjective health status. Work-related characteristics included work type (three-shift rotation v. others), current hospital work experience, workload, type of nursing unit, and hospital size (beds). Workload, as developed by Brewer et al. [29], is measured as the level of performance required for a job in terms of the amount, intensity, and frequency of work [30]. It comprised four items with a six-point Likert scale (“never” to “five or more days a week”). The total score ranges from 4 to 24, with high scores representing higher levels of workload. The Cronbach’s alpha in this study was 0.83.

### Data Analysis

Data analysis was conducted using SAS 9.4 (SAS, Cary, NC, United States). The means and standard deviations of the dependent variables (job satisfaction, intent to leave, and burnout) and the main independent variables (nurse staffing, work hours, mandatory overtime, and turnover) were evaluated. The dependent variables were continuous variables. Except for mandatory overtime, the three independent variables (nurse staffing, work hours, and turnover) were used as continuous variables. Mandatory overtime was dichotomous, and the reference group comprised nurses who did not work mandatory overtime. Descriptive statistics for the covariates (nurse- and work-related characteristics) were obtained. Univariate analysis for each variable and a multivariable GEE including only the significant variables in the univariate analysis were used to examine the impact of nurse staffing, work hours, mandatory overtime, and turnover on nurse outcomes after controlling for covariates. GEEs were used to account for the clustering (nursing unit). Owing to the missingness of each variable, the total number of samples used in each analytic model varied.

### Ethics Statements

The Institutional Review Board of a university approved this study (no. ewha-202205-0005-01). All participants provided informed consent online. Permission to use the instruments was obtained from the respective authors.

## Results

### Participant Characteristics

Nurses’ job satisfaction was 2.46 (standard deviation [SD] = 0.56) points on average, and intent to leave was 3.75 (SD = 0.87) points on average (Table 1). The average burnout was 29.71 (SD = 5.35) points, and 87% reported moderate levels of burnout. On average, nurses took care of 12.46 (SD = 3.53) patients during a shift. The average work hours for a shift were 9.33 (SD = 1.00), and 40% reported working mandatory overtime during the last month.

**TABLE 1:** General characteristics of study variables (*N* = 264) (Nurse staffing, work hours, mandatory overtime, and turnover in acute care hospitals affect nurse job satisfaction, intent to leave, and burnout: a cross-sectional study, South Korea, 2024).

Variables	*n* (%)	Mean (SD)
Dependent variables		
Job satisfaction	*N* = 235	2.46 (0.56)
Intent to leave	*N* = 237	3.75 (0.87)
Burnout	*N* = 236	29.71 (5.35)
22 or lower (low)	30 (11.2)	
23–41 (moderate)	233 (87.3)	
42 or higher (high)	4 (1.5)	
Main independent variables		
Nurse staffing level (number of patients per nurse during a shift)	*N* = 253	12.46 (3.53)
Work hours per shift	*N* = 231	9.33 (1.00)
Mandatory overtime	*N* = 253	
Yes	102 (40.3)	
No	151 (59.7)	
Nurse turnover rate for 6 months	*N* = 217	15.49 (14.20)
Covariates (nurse-and work-related characteristics)		
Sex	*N* = 234	
Male	5 (2.1)	
Female	229 (97.9)	
Age, years in 2022	*N* = 231	32.58 (7.97)
21–30	121 (52.4)	
31–40	67 (29.0)	
41–50	32 (13.8)	
≥51	11 (4.8)	
Highest nursing education	*N* = 230	
Associate degree	41 (17.8)	
Bachelor’s degree	178 (77.4)	
Master’s degree or PhD in nursing	11 (4.8)	
Marital status	*N* = 233	
Married or in domestic partnership	90 (38.6)	
Widowed, divorced, separated	5 (2.2)	
Never married	138 (59.2)	
Subjective health status	*N* = 231	
Very good	20 (8.7)	
Good	57 (24.7)	
Fair	111 (48.0)	
Poor	43 (18.6)	
Work type	*N* = 264	
3-shifts rotation	239 (90.5)	
Other	25 (9.5)	
Work experience in current hospitals (years)	*N* = 234	7.62 (7.18)
Under 1 year	16 (6.8)	
1 year–under 3 years	54 (23.1)	
3 years–under 5 years	37 (15.8)	
5 years–under 10 years	60 (25.6)	
10 years or over	67 (28.6)	
Workload	*N* = 263	18.05 (3.60)
Type of unit	*N* = 264	
Medical	77 (29.2)	
Surgical	97 (36.7)	
Medical-surgical	90 (34.1)	
Hospital size (beds)	*N* = 264	372.65 (115.03)
201–300	106 (40.2)	
301–400	49 (18.6)	
401–500	83 (31.4)	
501–1000	26 (9.8)	

Note. SD, standard deviation; PhD, Doctor of Philosophy.

Most participants were female (97.9%), and nurses’ mean age was 32.58 (SD = 7.97) years. More than 82% of the nurses had a bachelor’s degree or higher in nursing, and 59.2% had never been married. Regarding subjective health status, 18.6% reported poor health. More than 90% worked in a three-shift rotation, and they had 7.62 (SD = 7.18) years of work experience in their current hospital. The average workload was 18.05 (SD = 0.83) points. Approximately 34% of participants worked in medical-surgical units, and 40.2% worked in hospitals with 201–300 beds.

### Impact of Nurse Staffing, Work Hours, Mandatory Overtime, and Turnover on Job Satisfaction, Intent to Leave, and Burnout

Univariate and multivariable GEEs were used to examine the impact of nurse staffing, work hours, mandatory overtime, and turnover on job satisfaction, intent to leave, and burnout. In the univariate analysis, nurse staffing levels and turnover rates were significantly related to job satisfaction (Table 2). In the multivariable GEE, both nurse staffing (β = −0.037, standard error [SE] = 0.010) and turnover (β = −0.006, SE = 0.003) remained significant factors affecting job satisfaction. Job satisfaction decreased when nurses took care of more patients and worked in nursing units with greater turnover. Better health conditions increased job satisfaction; however, high workload levels decreased job satisfaction.

**TABLE 2:** Nurse staffing, work hours, mandatory overtime, turnover, and job satisfaction (Nurse staffing, work hours, mandatory overtime, and turnover in acute care hospitals affect nurse job satisfaction, intent to leave, and burnout: a cross-sectional study, South Korea, 2024).

Variables	β (SE)	*P*	β (SE)	*p*
Intercept			3.245 (0.237)	<0.001
Main independent variables				
Nurse staffing	−0.038 (0.010)	<0.001	−0.037 (0.010)	<0.001
Work hours	−0.072 (0.038)	0.059		
Mandatory overtime (ref: No)	−0.119 (0.075)	0.113		
Nurse turnover	−0.006 (0.003)	0.022	−0.006 (0.003)	0.015
Covariates (nurse-and work-related characteristics)				
Sex (ref: Female)				
Male	0.192 (0.255)	0.452		
Age, years in 2022	−0.007 (0.005)	0.144		
Highest nursing education (ref: Associate degree)				
Bachelor’s degree	−0.059 (0.097)	0.546		
Master’s degree or PhD in nursing	0.130 (0.190)	0.493		
Marital status (ref: Married or in domestic partnership)				
Widowed, divorced, separated	−0.303 (0.254)	0.231		
Never married	0.120 (0.075)	0.110		
Subjective health status (ref: Poor)				
Very good	0.328 (0.148)	0.027	0.379 (0.150)	0.012
Good	0.443 (0.111)	<0.001	0.402 (0.112)	<0.001
Fair	0.250 (0.098)	0.011	0.192 (0.010)	0.049
Work type (ref: Other)				
3-shift rotation	0.011 (0.126)	0.928		
Work experience in current hospitals	−0.004 (0.005)	0.458		
Workload	−0.039 (0.010)	<0.001	−0.026 (0.011)	0.014
Type of unit (ref: Medical-surgical)				
Medical	0.042 (0.093)	0.648		
Surgical	0.103 (0.087)	0.234		
Hospital size (ref: 201–300)				
301–400	0.090 (0.103)	0.381		
401–500	−0.159 (0.086)	0.064		
501–1000	−0.101 (0.125)	0.420		
*N*			187	

Note. SE, standard errors; PhD, Doctor of Philosophy.

Regarding intent to leave, nurse staffing and mandatory overtime were significant factors in the univariate analysis (Table 3). However, in the multivariable GEE, only mandatory overtime (β = 0.395, SE = 0.114) was significantly related. Compared to nurses who did not work mandatory overtime, those working mandatory overtime reported higher levels of intent to leave. Similar to the job satisfaction model, better health conditions decreased intent to leave, and workload increased intent to leave.

**TABLE 3:** Nurse staffing, work hours, mandatory overtime, turnover, and intent to leave (Nurse staffing, work hours, mandatory overtime, and turnover in acute care hospitals affect nurse job satisfaction, intent to leave, and burnout: a cross-sectional study, South Korea, 2024).

Variables	β (SE)	*p*	β (SE)	*p*
Intercept			3.203 (0.347)	<0.001
Main independent variables				
Nurse staffing	0.032 (0.016)	0.039	0.019 (0.016)	0.212
Work hours	0.102 (0.058)	0.076		
Mandatory overtime (ref: No)	0.399 (0.114)	<0.001	0.395 (0.114)	<0.001
Nurse turnover	0.003 (0.004)	0.557		
Covariates (nurse-and work-related characteristics)				
Sex (ref: Female)				
Male	0.041 (0.391)	0.916		
Age, years in 2022	−0.006 (0.007)	0.421		
Highest nursing education (ref: Associate degree)				
Bachelor’s degree	0.194 (0.148)	0.190		
Master’s degree or PhD in nursing	−0.417 (0.290)	0.150		
Marital status (ref: Married or in domestic partnership)				
Widowed, divorced, separated	−0.319 (0.389)	0.411		
Never married	−0.156 (0.115)	0.174		
Subjective health status (ref: Poor)				
Very good	−0.837 (0.224)	<0.001	−0.869 (0.221)	<0.001
Good	−0.692 (0.167)	<0.001	−0.684 (0.168)	<0.001
Fair	−0.545 (0.149)	<0.001	−0.530 (0.150)	<0.001
Work type (ref: Other)				
3-shift rotation	0.328 (0.192)	0.088		
Work experience in current hospitals	−0.006 (0.008)	0.440		
Workload	0.058 (0.015)	<0.001	0.035 (0.016)	0.028
Type of unit (ref: Medical-surgical)				
Medical	−0.081 (0.142)	0.571		
Surgical	−0.050 (0.133)	0.708		
Hospital size (ref: 201–300)				
301–400	−0.007 (0.160)	0.967		
401–500	0.126 (0.133)	0.343		
501–1000	−0.025 (0.194)	0.897		
*N*			212	

Note. SE, standard errors; PhD, Doctor of Philosophy.

Nurse staffing, work hours, mandatory overtime, and turnover were not significantly related to burnout (Table 4). Univariate analysis revealed that sex, age, education level, subjective health status, workload, and hospital size were significantly associated with burnout. In the multivariable GEE, only subjective health status and workload were significantly related to burnout. Compared to nurses with poor health conditions, nurses with very good (β = −0.790, SE = 0.128), good (β = −0.602, SE = 0.094), and fair (β = −0.368, SE = 0.082) health conditions reported lower burnout. Increased levels of workload led to greater levels of burnout (β = 0.028, SE = 0.009).

**TABLE 4:** Nurse staffing, work hours, mandatory overtime, turnover, and burnout (Nurse staffing, work hours, mandatory overtime, and turnover in acute care hospitals affect nurse job satisfaction, intent to leave, and burnout: a cross-sectional study, South Korea, 2024).

Variables	β (SE)	*p*	β (SE)	*p*
Intercept			3.119 (0.221)	<0.001
Main independent variables				
Nurse staffing	0.013 (0.010)	0.188		
Work hours	0.058 (0.037)	0.114		
Mandatory overtime (ref: No)	0.055 (0.072)	0.442		
Nurse turnover	0.001 (0.003)	0.629		
Covariates (nurse-and work-related characteristics)				
Sex (ref: Female)				
Male	−0.484 (0.240)	0.044	−0.233 (0.206)	0.258
Age, years in 2022	−0.011 (0.004)	0.016	−0.006 (0.004)	0.114
Highest nursing education (ref: Associate degree)				
Bachelor’s degree	−0.038 (0.092)	0.678	−0.114 (0.079)	0.148
Master’s degree or PhD in nursing	−0.364 (0.180)	0.042	−0.157 (0.161)	0.330
Marital status (ref: Married or in domestic partnership)				
Widowed, divorced, separated	−0.088 (0.247)	0.722		
Never married	0.013 (0.073)	0.856		
Subjective health status (ref: Poor)				
Very good	−0.914 (0.125)	<0.001	−0.790 (0.128)	<0.001
Good	−0.710 (0.093)	<0.001	−0.602 (0.094)	<0.001
Fair	−0.409 (0.083)	<0.001	−0.368 (0.082)	<0.001
Work type (ref: Other)				
3-shift rotation	0.153 (0.120)	0.198		
Work experience in current hospitals	−0.008 (0.005)	0.106		
Workload	0.040 (0.009)	<0.001	0.028 (0.009)	0.001
Type of unit (ref: Medical-surgical)				
Medical	0.071 (0.088)	0.420		
Surgical	−0.041 (0.082)	0.617		
Hospital size (ref: 201-300)				
301–400	0.066 (0.099)	0.508	0.020 (0.090)	0.828
401–500	0.081 (0.082)	0.322	0.033 (0.071)	0.639
501–1000	0.241 (0.119)	0.044	0.196 (0.106)	0.065
*N*			224	

Note. SE, standard errors; PhD, Doctor of Philosophy.

## Discussion

This study investigated the impact of nurse staffing, work hours, mandatory overtime, and turnover on nurse outcomes, including job satisfaction, intent to leave, and burnout. The mean score of nurses’ job satisfaction was 2.46, which is between “dissatisfaction (2 points)” and “satisfaction (3 points).” The average score for intent to leave was 3.75 points, which was 15.00 points in the total score; a high proportion of nurses reported that they wanted to leave their positions. The average burnout score (29.71 points) was higher than previously reported (27.49 points) [25]. Compared to a previous study [30], nurses in this study took care of fewer patients per shift and worked more than 9 h per shift, which is similar to the nurses in the previous study with a higher proportion working mandatory overtime (40%). Their turnover rate (15.49 for 6 months) was significantly higher than the national annual average (15.2%) [8].

Factors contributing to nurse outcomes differed among nurse staffing, work hours, mandatory overtime, and turnover. Job satisfaction was affected by both nurse staffing and turnover. A meta-analysis [14] reported that when the number of patients per nurse increased, nurses’ job dissatisfaction increased significantly (odds ratio = 1.08). The current findings support this relationship. Regarding turnover, a Canadian study [17] found that the 1-year turnover rate decreased nurses’ job satisfaction, which coincides with this study. Furthermore, the 6 months turnover rate decreased job satisfaction. Nurse staffing and turnover should be improved to increase job satisfaction.

The implications of these findings are that individual hospitals should make efforts to improve nurse staffing levels. Simultaneously, nurse staffing policies should be implemented at the state and national levels. For example, the United States has implemented several nurse staffing policies, including mandating minimum nurse staffing ratios in California, a staffing committee requiring a high proportion of registered nurses as members, and public disclosure of nurse staffing levels [31]. During the COVID-19 pandemic, several states adopted nurse staffing policies [32, 33]. The findings support policy changes aimed at improving nurses’ job satisfaction. Other countries should also adopt and implement nurse staffing policies.

Regarding intent to leave, mandatory overtime was the only significant factor contributing to intent to leave, although nurse staffing was significant in the univariate analysis, which differed from a previous meta-analysis [14]. Mandatory overtime increases the incidence of musculoskeletal disorders, injuries, and illnesses [16]. It did not have a relationship with intent to leave in a South Korean study [30]. In this study, working mandatory overtime increased intent to leave, which might indicate that a coercive working culture (working mandatory overtime) led to a higher intent to leave. Further studies are needed to investigate this relationship. Mandatory overtime should be prohibited to reduce nurses’ intent to leave, and exceptional conditions of mandatory overtime need to be evaluated for appropriateness.

Regarding policy implications, mandatory overtime has been banned in several states in the United States [12]. For example, Washington does not allow employees of healthcare facilities to work overtime, and the acceptance of working overtime among employees should be voluntary [34]. A national study found that policies banning mandatory overtime reduce the likelihood of mandatory overtime [35]. The relationship between mandatory overtime and intent to leave found in this study can be used as empirical evidence to expand this state policy to ban mandatory overtime among nurses.

None of the main independent variables were significant factors contributing to burnout, which differs from prior findings [14, 16]. Subjective health status and workload significantly affected burnout. These two factors also affect job satisfaction and the intent to leave. Based on the current findings, subjective health conditions should be promoted. Health promotion programs should be implemented, and their effectiveness should be evaluated. Concurrently, working conditions that may be harmful to nurses’ health should also be improved. Individual nurses and organizational efforts should be made to improve health conditions. Regarding workload, a previous South Korean study also found a significant relationship between job satisfaction and intent to leave in the expected direction [30]. A previous study [36] found that workload can increase burnout. The current findings support the relationship between workload and nurse outcomes. Workloads reported by nurses should be measured, monitored, and managed to improve nurse outcomes.

This study measured multiple aspects of sub-optimal nurse staffing (nurse staffing, work hours, mandatory overtime, and turnover), which is a key strength. However, the study has certain limitations. Because convenience sampling was used, the generalizability of the findings is lacking. Nurses who are more concerned about nurse outcomes and work conditions may respond to the survey, which could lead to self-selection bias. In addition, all data were collected from an online survey and were self-reported by nurses, which could create recall and socially desirable biases. Furthermore, the use of a cross-sectional design limits the ability to infer causal relationships among nurse staffing, work hours, mandatory overtime, turnover, and nurse outcomes. The calculation of nurse staffing levels, determined by the number of beds per the total number of nurses multiplied by 4.8, was based on the assumption that staffing levels remain consistent across shifts, workdays and weekends. However, this assumption holds true in only a few cases, which might be another limitation. Additionally, the work hours per shift, not the total work hours, measured in full time equivalent were used in this study. This omitted variable might affect the findings. Finally, mandatory overtime was measured by nurses’ perception of mandatory overtime. However, even without mandatory overtime, peer pressure might create a negative culture around working time. In this case, the actual value might be under estimated. These limitations should be addressed in future longitudinal studies.

### Conclusion

This study investigated the effect of sub-optimal nurse staffing on nurse outcomes. Sub-optimal nurse staffing and nurse outcomes were assessed using multifaceted variables. The study found a significant impact of nurse staffing, mandatory overtime, and turnover on job satisfaction and intent to leave. Subjective health conditions and workload affect job satisfaction, intent to leave, and burnout.

Nurse staffing policies and improvement programs in hospitals, as well as state- and national-level policy changes should be implemented to improve nurses’ job satisfaction. Similarly, mandatory overtime should be prohibited and working overtime among employees should be voluntary. Labor policy should ban mandatory overtime among nurses, which can improve their intent to leave.

## Data Availability

The data that support the findings of this study are available from the corresponding author upon reasonable request.
